# Ethnicity and suicide attempt: analysis in bipolar disorder and schizophrenia

**DOI:** 10.1186/1471-244X-13-252

**Published:** 2013-10-08

**Authors:** Ali Bani-Fatemi, Gina Polsinelli, James L Kennedy, Vincenzo De Luca

**Affiliations:** 1 Group for Suicide Studies, CAMH, Department of Psychiatry, University of Toronto, 250 College St., M5T 1R8 Toronto, ON, Canada; 2 Neurogenetics, CAMH, Department of Psychiatry, University of Toronto, Toronto, ON, Canada

**Keywords:** Ethnicity, Suicide attempt, Schizophrenia, Bipolar disorder, Schizoaffective disorder

## Abstract

**Background:**

Evidence is mixed as to whether White Europeans are at a higher risk for suicide attempts or completions compared to other ethnic groups. The present analysis assessed whether risk for suicide attempt was associated with White European ethnicity in 907 subjects with schizophrenia or bipolar disorder.

**Methods:**

Subjects were diagnosed using the Structured Clinical Interview for DSM-IV, and ethnicity was determined by self-report. Subjects were recruited from psychiatric care centers in Toronto, Canada. Logistic regression correcting for clinical covariates like age, gender and diagnosis, was used in this study.

**Results:**

We found no difference in suicide attempter status in white and non-white subjects who were diagnosed with schizophrenia and bipolar disorder.

**Conclusion:**

Our study does not support the evidence that White-European patients in North America are at higher risk for suicide attempt compared to non-European descent subjects. However, this result has to be replicated in larger studies in patients with these disorders.

## Background

Suicide attempt and suicide are a major concern in bipolar disorder (BD) and schizophrenia (SCZ). Up to 50% of patients with BD [1] and about 30% of patients with SCZ will attempt suicide in their lifetime, with slightly higher rates found in schizoaffective disorder (SZA) than SCZ [2]. Although more patients with mood disorders may attempt suicide than those with other psychiatric disorders ([1,3,4]), suicide can be seen as an independent heritable trait which can be assessed across diagnoses [4]. It is well established that previous suicide attempts are the strongest indicator for suicide completion ([3,5,6]), but speculations of risk factors for suicide attempts such as ethnicity in psychiatric populations have been more uncertain. The present analysis assessed whether risk for suicide attempt was associated with ethnicity in subjects with SCZ and BD.

Suicide in Canada remains a concern as 2611 individuals died by suicide in 2007 alone; about 10.2 per 100,000 Canadians [7]. Although consistent national data on ethnicity and completed suicide are unavailable in Canada, in the United States, recent Data from the National Center for Injury Prevention and Control (NCIPC) indicates that white Caucasians of European decent were much more likely than Blacks or Hispanics to commit suicide [8]. For every 100, 000 deaths, about 15 were from suicide in white individuals- more than double of the suicide rates in Black and Hispanic groups. However, studies have yielded mixed findings when studying suicide in SCZ, BD or other psychiatric populations, compared to the general population. For example, Montros and colleagues (2008) [9] conducted an analysis of suicide attempts in schizophrenia-spectrum disorders, and found that ethnicity was not a risk indicator for suicide attempts or for current suicidality. Another investigation by Borges and colleagues (2011) [10] of 15,180 subjects in a group of cross-sectional surveys found that after correcting for psychiatric diagnosis, there was no difference in suicide attempt in white Caucasian, Hispanic, Black or Asian individuals, although White subjects had higher rates of suicide ideation. Contradictory to data from the NCIPC, Bhui and colleagues (2011) [11] found in a group of psychiatric inpatients, that men from black African background were more likely than white British men to commit suicide, but that usual indicators of suicidal behavior- suicidal ideas, depressive symptoms, emotional distress, and hopelessness - were significantly more common in White British inpatients. Additional studies have found higher rates of suicide among black Caribbean and black African men than other ethnic groups who had been in contact with mental health services [12]. When considering women who had committed suicide as inpatients, it was found that they were significantly more likely to be of an ethnic minority group (Caribbean, South Asian) than white British women [11]. However, these findings were from UK groups and this evidence may be limited to this region. The question remains which ethnic groups, in individuals with BD and SCZ specifically, are at higher risk for suicide. Since suicide attempts remain the best predictor of suicide completion, we have used this as our main outcome.

The overall sample included detailed information on ethnicity and suicide attempts for 907 subjects with SCZ, BD and SZA. The current study is a secondary analysis of a clinical sample collected for a molecular genetics study.

We collected demographic and clinical data related to suicide attempts and corrected for these covariates in our analysis, such as age, gender, diagnosis, duration of illness and a history of alcohol or substance abuse. Separate statistical analyses were conducted to assess whether ethnicity is associated with suicide attempts across patient groups, or whether results are different when assessing each patient group separately.

## Methods

### Subjects

For the aim of this study, we used two samples: one with diagnoses of bipolar spectrum disorder (Table 1) and one with schizophrenia spectrum disorder (Table 2).

**Table 1:** Subject demographic and diagnostic information for subjects with bipolar disorder

	**All**	**Suicide Attempters**	**Non-Attempters**
Bipolar* (N)	341	74	267
White** (N)	305	62	243
**Age (X, SD)**	35.64+/-10.54	36.06+/-10.30	35.54+/-10.62
**Females (N): Males (N)**	209:132	45:29	164:103
**AAO (X, SD)**	20.22,+/- 7.65	19.78+/-8.88	20.34+/-7.38
**DOI (X,SD)**	17.67,+/-11.06	19.40+/-11.11	17.21+/-11.02
**Substance abuse (N)**	24	8	16
**Alcohol abuse (N)**	69	18	51
**Family History (N)**	275	53	222
**Psychotic Sx (N)**	173	36	137
**Axis-I Comorbidity**	84	21	63

Demographic and diagnostic characteristics by life time suicide attempters and non-attempters in bipolar subjects. AAO = Age at Onset, DOI = Duration of illness.

*Subjects with SZA bipolar type were grouped with BD subjects in the statistical analyses.

**White refers to White Europeans.

**Table 2:** Subject demographic and diagnostic information for subjects with schizophrenia

	**All**	**Suicide Attempters**	**Non-Attempters**
Schizophrenia* (N)	566	192	374
White** (N)	401	145	256
**Age (X, SD)**	39.28+/-11.45	41.34 +/- 11.06	38.23+/-11.52
**Females (N): Males (N)**	179:387	72:120	107:267
**AAO (X, SD)**	21.79+/-6.66	21.40 +/- 6.20	21.99+/-6.91
**DOI (X, SD)**	17.46 +/-11.43	19.92 +/- 11.11	16.18+/-11.41
**Substance abuse (N)**	266	99	167
**Alcohol abuse (N)**	174	62	112
**Family History (N)**	232	94	138
**Tobacco Use (N)**	311	109	202
**Affective (N)**	58	34	24
High Hospitalizations (N)***	266	120	146

Demographic and diagnostic characteristics in life time suicide attempters and non-attempters in subjects with SCZ. AAO = Age at Onset, DOI = Duration of illness. *Subjects with schizoaffective depressive type were grouped with those with schizophrenia in analyses. **White refers to White Europeans. ***Dichotomized according to the median in our sample (median = 4); 4 and above is considered high number of hospitalization.

The inclusion criteria were a diagnosis of SCZ, SZA or BD according to DSM-IV using the Structured Clinical Interview for DSM-IV (SCID-I/P). Patients were recruited from several psychiatric care centers in Toronto, and were interviewed by an experienced researcher to assess lifetime suicide attempt. Of the 907 subjects in this analysis, 710 were White European and 197 were non White European; see Table 3 for a detailed breakdown of subtypes, subject demographic characteristics and clinical variables. Subjects were mostly unrelated; however, there were few related individuals (first degree relatives) who were recruited independently.

**Table 3:** Subject demographic and diagnostic information for all subjects

	**All Subjects**	**Suicide Attempters**	**Non-Attempters**
**Total (N%)**	**907**	**266 (29.33%)**	**641 (70.67%)**
White* (N%)	**710**	**209 (29.44%)**	**501 (70.56%)**
**Age (X, SD)**	**37.94+/-11.26**	**39.89 +/- 11.09**	**37.13 +/-11.23**
**Females: Males (N%)**	**388:519**	**117 (43.98%):149 (56.02%)**	**271 (42.28%):370 (57.72%)**
**AAO (X, SD)**	**21.20 +/- 7.09**	**20.94 +/- 7.03**	**21.30+/-7.10**
**DOI (X,SD)**	**17.53+/-11.30**	**19.79 +/- 11.08**	**15.68+/-11.27**
**Substance abuse (N%)**	**266**	**99 (37.22%)**	**167 (62.78%)**
**Alcohol abuse (N%)**	**243**	**80 (32.92%)**	**163 (67.08%)**
**Family History (N%)**	**507**	**147 (28.99%)**	**360 (71.01%)**
**Psychotic Sx (N%)**	**744**	**229 (30.78%)**	**515 (69.22%)**
**Affective Sx (N%)**	**398**	**108 (27.14%)**	**290 (72.86%)**

Demographic and diagnostic characteristics by life time suicide attempters and non-attempters. AAO = Age at Onset. DOI = Duration of illness. *White refers to White Europeans.

### Assessment

Demographic and clinical variables were collected in a cross-sectional interview by a clinician or trained research assistant. The following demographic and clinical variables were collected using the Structured Clinical Interview for DSM-IV (SCID-I/P) and the Family Interview for Genetic Studies (FIGS): demographic characteristics, age at onset (AAO), diagnostic subtype and the presence of affective and/or psychotic symptoms, duration of illness (DOI), number of hospitalizations, alcohol abuse and substance abuse. Other axis I comorbidity was not assessed in the schizophrenia sample and therefore was not included as covariate in the analysis. Affective symptoms were determined as present in both SZA subtypes and in BD. Psychotic symptoms were coded present in all SCZ and SZA patients, and in BD patients who presented with psychotic symptoms (i.e. auditory/visual hallucinations or delusions, or both). A cross-sectional assessment was used to obtain the information on suicide attempt and suicidal behavior lifetime to ascertain the presence of suicide attempt using the structured interview for DSM-IV and collecting a comprehensive life chart and then patients were categorized in two groups: attempters if they attempt at least once during their life and non-attempters if they never attempt suicide during their life.

In this study we considered region of origin and ancestry as two aspects of ethnicity. In this regard, the subjects were asked where their family (both maternal and paternal grandparents) came from, and their origin was collected by self-report. Then, each subject was categorized as “White European” when all four grandparents were reported as White Europeans or “Non white European” when the grandparents were coming from different backgrounds.

The Research Ethics Board of the Centre for Addiction and Mental Health (CAMH) reviewed and approved the study. Written informed consent was obtained from all participating subjects.

### Statistical analysis

Binary logistic regression analyzed the risk of being a suicide attempter using ethnicity as our dichotomous main predictor variable -White European versus all other ethnicities- to test the hypothesis that White European individuals attempt suicide more than other ethnicities. The presence of a suicide attempt lifetime was used as binary dependent variable (attempters versus non-attempters).

Three separate analyses were conducted with the same statistical method and hypothesis, and clinical covariates related to risk for suicide were included. Firstly, an analysis including all subjects was conducted to assess the effect of ethnicity considering suicide attempt as a trait across diagnoses (Table 3). Secondly, an analysis including patients with bipolar spectrum diagnosis only (BD and SZA bipolar type) was conducted including simultaneously sex, subtype (BDI, BDII), duration of illness (DOI), the presence of alcohol abuse lifetime, the presence of substance abuse lifetime, psychotic features, axis I co-morbidity and family history of mood disorders, in addition to ethnicity (Table 1). Sex, presence of substance/alcohol abuse, psychotic features and duration of illness were included as covariates, since they are well known risk modifiers for suicidal behavior. Subtype, axis I co-morbidity and family history were included since they are associated with the severity of the illness.

Thirdly, the same analysis in patients with schizophrenia spectrum diagnosis (SCZ and SZA depressive type) was conducted with all covariates together with ethnicity: sex, DOI, presence of affective symptoms, number of hospitalizations, family history of psychiatric disorders, the presence of alcohol abuse lifetime, the presence of substance abuse lifetime and ethnicity (Table 2). A re-analysis excluding the related subjects was performed to test whether limited violation of assumption of independent observations made any difference.

## Results

Table 3 shows the demographic characteristics of the suicide attempters (n = 266) and non-attempters (n = 641). The sample consists of 519 males and 388 females, and of the 907 subjects in this analysis, 710 were White European and 197 were not White European. In our analysis entering all variables and including all subjects, duration of illness (p < 0.001), diagnosis of schizophrenia spectrum disorder (p = 0.003), substance abuse (p = 0.009) and female gender (p = 0.031) were significantly associated with suicide attempt (Table 4). Ethnicity was not significantly associated with suicide attempt in the overall sample (OR = 1.043; CI 95% = 0.713-1.524, p = 0.830) (Table 4).

**Table 4:** Statistical analysis results: analysis of predictors of suicide attempt (all subjects)

	**Wald**	**df**	**Sig.**	**OR**	**95.0% C.I. for OR**	
Ethnicity	.046	1	0.830	1.043	.713	1.524
Sex	4.663	1	0.031	.699	.505	.967
DOI	13.959	1	0.000	1.026	1.012	1.040
Substance Abuse**	6.889	1	0.009	1.598	1.126	2.268
Alcohol Abuse**	.744	1	0.388	1.013	.983	1.045
Diagnosis*	8.808	1	0.003	.574	.398	.828

Results for binary logistic regression analysis testing each of the variables simultaneously. DOI = Duration of illness. *Schizophrenia spectrum versus Bipolar Spectrum; **Lifetime variable.

In the analysis including SCZ and SZA (depressive type only), female gender, high number of hospitalizations, family history of psychiatric disorders and presence of affective symptoms were significantly associated with suicide attempt when all the clinical variables were included simultaneously in the model (Table 5). However, ethnicity was not statistically significant (p = 0.973) (Table 5). Furthermore, we removed six related subjects and the five variables associated with suicide attempt did not lose significance.

**Table 5:** Analysis in schizophrenia spectrum patients*

	**Wald**	**df**	**Sig.**	**OR**	**95.0% C.I. for OR**	
Ethnicity	.001	1	.973	1.009	.590	1.726
Sex	6.090	1	.014	.531	.322	.878
DOI	.180	1	.671	1.006	.980	1.031
Substance Abuse**	5.043	1	.025	1.742	1.073	2.827
Alcohol abuse**	.197	1	.658	1.021	.932	1.119
Family history of psychiatric disorders	7.571	1	.006	1.906	1.204	3.017
Affective Symptoms	11.857	1	.001	5.329	2.056	13.813
High number of Hospitalization***	9.373	1	.002	2.324	1.354	3.987

Results for binary logistic regression analysis including all variables together. DOI = duration of illness. *Includes SZA depressive type. **Lifetime variable. ***The threshold was four or more hospitalizations since four was the median of the number of hospitalizations.

In the final analysis including only BD spectrum patients, no clinical covariates were associated with suicide attempt when analyzed together (Table 6) and ethnicity was not significant (OR = 0.609, 95% CI: 0.225-1.646, p = 0.329). We also removed 62 related subjects and there was no change in the significance of the all covariates.

**Table 6:** Analysis in bipolar disorder*

	**Wald**	**df**	**Sig.**	**OR**	**95.0% C.I.for OR**	
Ethnicity	.995	1	.329	.609	.225	1.646
**Sex**	.006	1	.939	.974	.502	1.893
DOI	1.171	1	.279	1.015	.988	1.043
**Substance** Abuse***	.652	1	.419	1.557	.531	4.566
Alcohol Abuse***	1.160	1	.281	1.461	.733	2.910
Family history of Psychiatric Disorders	1.665	1	.197	.605	.282	1.298
**Psychotic Sx**	.674	1	.412	.774	.420	1.427
Axis-I Comorbidity	1.074	1	.300	1.441	.722	2.875
Subtype** (Schizoaffective)	2.294	1	.130	2.405	.773	7.488
Subtype** (Bipolar II)	1.135	1	.287	.681	.336	1.381

Results for binary logistic regression analysis including all variables together. DOI = duration of illness. *Includes schizoaffective bipolar type, **Reference category is Bipolar Type I, ***Lifetime variable.

## Discussion

The present analysis assessed whether ethnicity was associated with risk for suicide attempt in patients with SCZ, SZA and BD. Ethnicity was not associated with risk for suicide attempt in our analysis including all subjects, nor when considering individuals with BD, nor in our separate analysis considering SCZ. On the other hand, a number of other risk factors were significantly correlated with suicide attempts in SCZ, such as history of substance abuse and affective symptoms. However, in BD, no clinical variables were related to risk for suicide attempt.

Previous studies in subjects with psychiatric conditions or schizophrenia yielded mixed evidence as to whether ethnicity could be associated with suicidal behavior, although white ethnicity has been established as the highest risk group for suicide in literature [13] and US national data [8]. The present analysis tested the hypothesis that White European ethnicity would be more at risk for suicide attempts than other ethnicities, but our findings indicated no association with ethnicity.

Our results are akin to those found in other studies exclusively on individuals with schizophrenia-spectrum disorders [9], but are inconsistent with other recent studies and public health data. This may be because our main outcome was suicide attempt rather than suicide completion, which has been the main outcome in studies of ethnicity and suicidal behavior ([3,8,12]). Although suicide attempts remain the most reliable predictor of suicide completion ([3,5,6,14]) there may be differential risk factors between attempts and completions. In non-psychiatric populations, for example, lower socioeconomic status has been related to suicide attempts. However, in SCZ, the opposite relationship is found [15]. It is possible that in SCZ and BD, other factors related to these illnesses influence risk for suicide attempts, superseding any heritable or social factors related to their ethnicity. In SCZ these include severity of positive symptoms, hopelessness, frequent brief hospitalizations, co-morbid obsessive compulsive disorder and good insight to illness [16]. Considering that SCZ and BD are serious neuropsychiatric illnesses with potentially severe and debilitating consequences, factors related to these diseases may play a bigger role in risk for suicide attempts, which span across ethnicity. Thus, in the SCZ and BD populations, consistently with other studies, we found no relationship between ethnicity and suicide attempts and this might suggest that ethnicity should not be considered a major risk factor for suicide within these clinical groups.

A similar example of a well established risk factor which confers lower risk for suicide in SCZ than in the general population, is gender. In Canada, the ratio of male to female completed suicide is four to one, with this rate being similar in the United States and Western Europe [17]; except in SCZ where this gender difference of suicide completions is not as evident [16]. The same inconsistency may be true for ethnicity; although it is probably true that white ethnicities commit suicide more often in the general population, this may not be the case in major psychoses.

In addition, it is possible that ethnicity influences suicide attempt and psychiatric diagnosis independently. In an analysis of 6865 completed suicides from the National Violent Death Reporting System (NVDRS) from 2004, white Caucasians were more likely to be diagnosed with depression or BD and those identified as black were more likely to have schizophrenia [18]. To add confusion to these findings, and supporting the assumption that white ethnicity is at higher risk, Rocket and colleagues [19] found that Black and Hispanic suicide victims recorded in the US National Center for Health Statistics were more likely to be misclassified as suicide, giving inaccurate exacerbation of Black and Hispanic suicide victims.

Although some papers have found an association between black African and black Caribbean ethnicity and suicide, in psychiatric inpatients or patients in contact with mental health services [12], these studies have used samples from the United Kingdom, where ethnic minorities may have social conditions distinct from those in Canada. These conditions may have provided different environmental conditions conferring risk for suicide.

A major strength to this paper is the detailed classification of ethnicities, which enabled us to more precisely assess heritable contributions which may be ethnically-linked in conferring risk for suicide attempt. We aimed to eliminate results associated only with participant’s self-reported ethnicity, which could be based more on the socio-cultural identity than the ancestry. Previous studies have relied on subject self-reported ethnicity, or by retrospective review of ethnicity data from government sources which often use self-reported ethnicity. Analyses can be conducted in such a way to assess ethnicity more loosely, considering how subjects self-identify; however, to more accurately assess differences in ethnicity which are inherited among defined groups, our method is substantially more reliable. On the other hand, differences within the White European group were not analyzed and an imbalance among the several European ancestries included in the White European group may explain the inconsistency with previous studies. Furthermore, we did not have access to the immigration history for our study subjects and this could have been a confounding factor in our group analysis, since migration is conferring risk for suicide [20] and suicide attempt [21].

An additional advantage to this analysis is that we were able to interview all participants to collect ethnicity and the structured interview for DSM-IV was used for the diagnosis of our patients, rather than relying on clinician judgment or reviewing medical records retrospectively. Thus our analysis investigated the relationship between ethnicity and suicide attempt in clearly defined psychiatric and ethnic groups, whereas many previous analyses have mixed or unclear samples which are unable to elucidate this relationship in these patient groups exclusively.

However, one limitation of this study is the possibility that we did not reject a false null hypothesis that white and non-white have the same rate of suicide attempt because we did not have enough statistical power.

Despite these limitations, such as lack of migration history and heterogeneity of ethnicity, this study confirm important indicators for clinicians hoping to identify patients at risk for suicide attempts with SCZ and BD.

## Conclusion

Although we found no association between ethnicity and suicide attempt, our sample did show that other factors such as female gender, substance abuse, a high number of hospitalizations, diagnosis of schizoaffective disorder and family history of psychiatric disorders may be associated with an increased risk for suicide attempts in schizophrenia spectrum disorders. Furthermore, the association between longer duration of illness and suicide attempt lifetime was found in the total sample and likely reflects a broader exposure window in which this outcome may be observed.

Future studies may compare the difference in suicide attempts between interviewer determined ethnicity (collected as part of the family history) versus an individual’s self-reported ethnicity to examine the difference between social and inherited factors. In addition, data from suicide attempts and suicide completions can be compared to see if there is a direct relationship in suicide completion rates and suicide attempt rates in different ethnic groups. Such a study should include measures related to suicide attempts such as symptom severity, hopelessness, and number of hospitalizations as factors that clinicians can take into account when assessing for suicide risk.

## Competing interests

The authors declare that they have no competing interest.

## Authors’ contributions

GP and AB-F contributed equally to this paper. GP wrote the first version of the paper and performed the initial analysis. AB-F wrote the final version of the paper and performed the final analysis. JK is a senior collaborator. VDL is the Senior Responsible Author and designed the experiment. All authors read and approved the final manuscript.

## Pre-publication history

The pre-publication history for this paper can be accessed here:

http://www.biomedcentral.com/1471-244X/13/252/prepub

## References

[B1] Jamison KR Suicide and bipolar disorder J Clin Psychiatry 2000 13 Suppl 9 47 51 10826661

[B2] Radomsky ED Suicidal behavior in patients with schizophrenia and other psychotic disorders Am J Psychiatry 1999 13 10 1590 5 10518171 10.1176/ajp.156.10.1590

[B3] Alberdi-Sudupe J Suicide attempts and related factors in patients admitted to a general hospital: a ten-year cross-sectional study (1997–2007) BMC Psychiatry 2011 13 51 10.1186/1471-244X-11-51 21453478 PMC3078091

[B4] Mann JJ Neurobiology of suicidal behaviour Nat Rev Neurosci 2003 13 10 819 28 14523381 10.1038/nrn1220

[B5] Jenkins GR Suicide rate 22 years after parasuicide: cohort study Bmj 2002 13 7373 1155 10.1136/bmj.325.7373.1155 12433767 PMC133456

[B6] Oquendo MA Currier D Mann JJ Prospective studies of suicidal behavior in major depressive and bipolar disorders: what is the evidence for predictive risk factors? Acta Psychiatr Scand 2006 13 3 151 8 10.1111/j.1600-0447.2006.00829.x 16889585

[B7] Suicide and suicide rate, by sex and by age grouphttp://www.statcan.gc.ca/tables-tableaux/sum-som/l01/cst01/hlth66d-eng.htm

[B8] Suicide rates among persons ages 10 years and older, by race/ethnicityhttp://www.cdc.gov/violenceprevention/suicide/statistics/rates02.html

[B9] Montross LP Suicidal ideation and suicide attempts among middle-aged and older patients with schizophrenia spectrum disorders and concurrent subsyndromal depression J Nerv Ment Dis 2008 13 12 884 90 10.1097/NMD.0b013e31818ec823 19077855

[B10] Borges G Suicidality, ethnicity and immigration in the USA Psychol Med 2011 13 6 1175 84 22030006 10.1017/S0033291711002340 PMC3733100

[B11] Bhui KS Dinos S McKenzie K Ethnicity and its influence on suicide rates and risk Ethn Health 2011 13 1–2 141 8 22181826 10.1080/13557858.2011.645151

[B12] Bhui KS McKenzie K Rates and risk factors by ethnic group for suicides within a year of contact with mental health services in England and Wales Psychiatr Serv 2008 13 4 414 20 10.1176/appi.ps.59.4.414 18378841

[B13] Steele MM Doey T Suicidal behaviour in children and adolescents. part 1: etiology and risk factors Can J Psychiatry 2007 13 6 21S 33S 17824350

[B14] Marangell LB Prospective predictors of suicide and suicide attempts in 1,556 patients with bipolar disorders followed for up to 2 years Bipolar Disord 2006 13 5 Pt 2 566 75 17042830 10.1111/j.1399-5618.2006.00369.x

[B15] Siris SG Suicide and schizophrenia J Psychopharmacol 2001 13 2 127 35 10.1177/026988110101500209 11448086

[B16] Mamo DC Managing suicidality in schizophrenia Can J Psychiatry 2007 13 6 Suppl 1 59S 70S 17824353

[B17] Langlois S Morrison P Suicide deaths and suicide attempts Health Rep 2002 13 2 9 22 12743953

[B18] Karch DL Barker L Strine TW Race/ethnicity, substance abuse, and mental illness among suicide victims in 13 US states: 2004 data from the national violent death reporting system Inj Prev 2006 13 Suppl 2 ii22 ii27 17170166 10.1136/ip.2006.013557 PMC2563485

[B19] Rockett IR Race/ethnicity and potential suicide misclassification: window on a minority suicide paradox? BMC Psychiatry 2010 13 35 10.1186/1471-244X-10-35 20482844 PMC2891687

[B20] Whitlock FA Migration and suicide Med J Aust 1971 13 17 840 8 5117285

[B21] Westman J The influences of place of birth and socioeconomic factors on attempted suicide in a defined population of 4.5 million people Arch Gen Psychiatry 2003 13 4 409 14 10.1001/archpsyc.60.4.409 12695319

